# ROCK inhibitors alter brain proteomic profiles and tau phosphorylation in iPSC‐derived human neurons and mouse model of Alzheimer’s disease

**DOI:** 10.1002/alz.091474

**Published:** 2025-01-03

**Authors:** Roberto Collu, Elisa Giunti, Zheng Yin, Sarah Daley, Mei Chen, Qinqing Li, Peter J Morin, Richard Killick, Stephen T.C. Wong, Weiming Xia

**Affiliations:** ^1^ Boston University, Boston, MA USA; ^2^ Houston Methodist Research Institute, Houston, TX USA; ^3^ Bedford VA Healthcare System, Bedford, MA USA; ^4^ University of Edinburgh, Edinburgh, Edinburgh United Kingdom; ^5^ Boston University Chobanian & Avedisian School of Medicine, Boston, MA USA; ^6^ Institute of Psychiatry, Psychology and Neuroscience, King’s College London, London United Kingdom; ^7^ Geriatric Research Education & Clinical Center, VA Bedford Healthcare System, Bedford, MA USA; ^8^ Kennedy College of Sciences, University of Massachusetts Lowell, Lowell, MA USA

## Abstract

**Background:**

We aim to investigate efficacies of Ras homolog (Rho)‐associated kinases (ROCK) inhibitors on Alzheimer’s disease (AD) pathological proteins in human induced pluripotent stem cell (iPSC)‐differentiated human neurons and the P301S tau transgenic mouse model (PS19).

**Method:**

Quantitative liquid chromatography‐mass spectrometry (LC‐MS/MS) and targeted ELISA were implemented to investigate the effect of treatment with fasudil or its derivatives on the human neurons and brains from PS19 mice. We explored the efficacy of these ROCK inhibitors in reducing tau phosphorylation, and the brain proteomic profiles after their administration in mice.

**Result:**

We found a significant negative correlation between the brain levels of phosphorylated tau (pTau) and both fasudil and its metabolite hydroxyfasudil. Human neuronal culture exposed to selective ROCK inhibitors exhibited similar efficacies. Proteomic profiling of mice brains exposed to fasudil revealed the activation of the mitochondrial tricarboxylic acid (TCA) cycle and blood‐brain barrier (BBB) gap junction metabolic pathways.

**Conclusion:**

Our results provide evidence to support the development of ROCK inhibitors as AD therapeutics.

